# Apparent Talker Variability and Speaking Style Similarity Can Enhance Comprehension of Novel L2-Accented Talkers

**DOI:** 10.1177/00238309251390505

**Published:** 2025-12-15

**Authors:** Nicholas B. Aoki, Georgia Zellou

**Affiliations:** Department of Linguistics, University of California, Davis, USA

**Keywords:** Perceptual learning, talker variability, generalization, speaking style, speech perception

## Abstract

Certain studies report facilitatory effects of multiple-talker exposure on cross-talker generalization of L2-accented speech (often defined as greater comprehension of novel talkers). However, a confound exists in prior work: do multiple-talker exposure benefits stem from the greater number of talkers (numerosity) or greater phonological variability (heterogeneity)? This study examined how apparent talker variability and speaking style affect L2-accent adaptation, while keeping phonological variation as constant as possible across exposure conditions. L1-English participants transcribed sentences in noise for a single Mandarin-accented English talker in an exposure phase and a novel Mandarin-accented English speaker in a test phase (a control condition received no exposure). Although all exposure stimuli came from one speaker, half of the listeners who received exposure were led to believe that multiple talkers were present by shifting the F0 and formants of a subset of sentences. We find: (a) when the test talker produces casual speech, all critical conditions with exposure enhance generalization (i.e., greater comprehension of the test talker relative to control); (b) when the test talker produces hard-of-hearing-directed speech, there is no difference in transcription accuracy between the control and critical conditions; and (c) when the test talker produces casual speech, generalization is greatest when listeners are exposed to multiple apparent talkers, but only given speaking style similarity between exposure and test (i.e., when the exposure phase also presents casual speech). This work lends credence to numerosity accounts—given a minimal change in phonological variability, the illusion of multiple-talker exposure can facilitate cross-talker generalization of L2-accented speech.

## 1 Introduction

The number of second-language (L2) speakers has rapidly increased in recent decades (Graddol, 2003; Salomone, 2022), prompting burgeoning interest in the perception of L2-accented speech (Baese-Berk et al., 2020). Extensive work demonstrates that listening to L2-accented speech is initially more effortful, resulting in reduced intelligibility, greater cognitive load, and slower word recognition compared with first-language (L1) accented speech (Kato & Baese-Berk, 2022; McLaughlin & Van Engen, 2020; Porretta et al., 2016). Addressing this disparity, a growing area of research asks how listeners can be trained to improve their comprehension of speakers from diverse language backgrounds (Bent & Baese-Berk, 2021; Derwing et al., 2002).

Successful perceptual adaptation often hinges upon the type of exposure that listeners receive (Kleinschmidt & Jaeger, 2015). For example, talker-specific learning is generally a robust phenomenon—previous exposure to a particular L2-accented talker enhances subsequent processing for that same talker (Bent et al., 2009; Clarke & Garrett, 2004; Xie et al., 2018; cf. Bradlow et al., 2023). Cross-talker generalization, however, is not as consistent (Baese-Berk, 2018). What exposure properties boost processing for *novel* talkers?

A substantial body of work in the cognitive sciences has suggested that exposure to greater variability is helpful (and possibly even necessary) for generalization (Raviv et al., 2022). In work on L2-accent or dialect adaptation, this effect is typically realized as a benefit of multiple-talker exposure over single-talker exposure (Bradlow & Bent, 2008; Bradlow et al., 1997; Clopper & Pisoni, 2004; Lively et al., 1993). However, at least three concerns have been identified within this literature. First, several recent studies have been unable to consistently replicate the advantage of multiple-talker exposure (Bradlow et al., 2023; Xie et al., 2021). Second, even if earlier work is taken for granted, facilitatory effects of talker variability are often confounded by several explanations, such as numerosity and heterogeneity accounts (Raviv et al., 2022). Third, although talker variability (between-talker variation) has been widely studied, within-talker variation is underexplored in adaptation research (cf. Sumner, 2011), leaving the role of variation itself on generalization undertheorized.

Taken together, it remains unclear exactly how (or whether) variation mediates cross-talker generalization. This study reflects an initial step toward alleviating the three previously raised issues. More specifically, this work builds upon a seminal study by Bradlow and Bent (2008), leveraging the speech-transcription-in-noise paradigm to assess the role of apparent talker variability and speaking style on generalization of L2-accented speech.

The rest of the introduction is organized as follows. Section 1.1 first summarizes Bradlow and Bent (2008) and then reviews its lack of replication. Section 1.2 discusses the challenges of disentangling numerosity and heterogeneity accounts. Section 1.3 motivates how apparent talker variability (the simulation of multiple-talker exposure) might begin to resolve certain theoretical confounds. Section 1.4 explores the potential role of acoustic similarity and speaking style (a type of within-talker variation) on generalization, and Section 1.5 concludes by detailing the design and predictions of this study.

### 1.1 Is talker variability necessary for generalization? A review of Bradlow and Bent (2008) and subsequent replication difficulties

Early work on L2-accent adaptation claimed that exposure to talker variability is necessary for cross-talker generalization. Bradlow and Bent (2008) asked participants to transcribe sentences in noise in an exposure phase and a subsequent test phase. All listeners were tested on the same (novel) Mandarin-accented English speaker, but heard different exposure materials based on their assigned condition. The critical result was that generalization only occurred for the multiple-talker exposure condition, not for the single-talker exposure condition. In more concrete terms—relative to a control group (exposure to multiple L1-English speakers), transcription accuracy for the novel test talker only increased after exposure to *multiple* Mandarin-accented English talkers, not after exposure to a *single* Mandarin-accented English talker.

Contra Bradlow and Bent (2008), however, more contemporary work on L2-accent perception has found that multiple-talker exposure may not be strictly necessary for generalization (Bradlow et al., 2023; Xie et al., 2021; Xie & Myers, 2017).^1^ Xie et al. (2021) observed parallel results across two high-powered replication experiments—both single-talker and multiple-talker exposure enhanced transcription accuracy for a novel talker, with little evidence for any additional benefit of multiple-talker exposure. Xie et al. (2021) account for this lack of replication by highlighting several issues within Bradlow and Bent (2008), including relatively low statistical power and the presence of methodological confounds (p. e36–e37).

In the context of L2-accent perception, the *necessity* of multiple-talker exposure for generalization is a relatively extreme position that has become increasingly tenuous, given the replicability concerns surrounding Bradlow and Bent (2008). A more viable alternative might test a comparatively moderate hypothesis—that multiple-talker exposure can be *helpful* for generalization, given the appropriate circumstances. Indeed, besides Bradlow and Bent (2008), numerous studies in the cognitive sciences have found benefits of variability on generalization (Raviv et al., 2022), which seems to warrant an explanation. There are at least two distinct mechanisms that could potentially account for a talker variability advantage (numerosity and heterogeneity), but successfully teasing them apart remains a practical challenge.

### 1.2 The challenge of adjudicating between numerosity and heterogeneity accounts

As Raviv et al. (2022) have highlighted, multiple-talker exposure differs from single-talker exposure in at least two respects: (a) an increase in the number of talkers and (b) an amplification of phonological variability. Both numerosity and heterogeneity accounts expect talker variability to boost generalization, but attribute the effect to different properties of multiple-talker exposure.

According to a *numerosity account*, the higher number of talkers is what facilitates generalization, not the higher amount of phonological variation. If listeners only hear a single L2-accented talker initially, there might be some uncertainty about whether the phonological properties of exposure are idiosyncratic or characteristic of the overall accent, thus weakening generalization. However, those exposed to multiple L2-accented speakers are more likely to notice shared attributes (e.g., a consistent devoicing of word-final stop consonants, such that “seat” is intended as “seed”; Xie & Myers, 2017), implying that the phonological properties of the input are broad accent features, not merely talker-specific particularities. The development of an accent-specific (talker-general) mental model might promote stronger generalization, which should increase comprehension for novel talkers with the same L2-accent (e.g., applying what they have learned from exposure, listeners are more likely to recognize that a stimulus produced as “beet” is intended as “bead”).

A *heterogeneity account* claims that the greater phonological variation of multiple-talker exposure is what encourages generalization, not the larger number of talkers. L1- and L2-accents often diverge in cue weighting, and exposure to a more variegated stimulus set boosts the likelihood of learning which dimensions are relevant for distinguishing speech sounds (Apfelbaum & McMurray, 2011). L1-English and Catalan-accented English speakers, for instance, differ in their production of tense/lax vowel contrasts (e.g., /i/ in “seat” vs. /ɪ/ in “sit”)—whereas L1-English speakers rely more heavily on formant frequencies than vowel duration (Hillenbrand et al., 2000), Catalan-accented English speakers show the opposite trend (Cebrian, 2007). As an extreme case, if there was no phonological variation in exposure (e.g., presentation of the same tokens of “seat” and “sit” from one talker), then it would be unclear whether the formant frequency and duration patterns were specific to those particular tokens or reflective of a broader cue weighting strategy. By contrast, exposure to a diverse set of Catalan-accented tokens would enable listeners to recognize a consistent lack of differentiation in formant frequencies compared with vowel duration. Multiple-talker exposure magnifies phonological variation, given the ubiquity of talker-specific differences in speech production (e.g., due to physiological and/or social factors, individual speakers tend to produce vowels with their own unique pattern of formant frequencies; Xie & Jaeger, 2020). Leveraging this greater variation, listeners exposed to multiple L2-accented talkers are more likely to successfully shift their cue weighting, which can then facilitate understanding of a novel talker from the same language background.

Numerosity and heterogeneity accounts are not intractably confounded, but teasing them apart is complicated in practice, especially in experiments on L2-accent adaptation that employ naturalistic, sentence-length stimuli (Bradlow & Bent, 2008; Xie et al., 2021). For one, speakers do not possess control over their exact degree of variation across utterances (Smith & Kenney, 1994), so researchers often cannot investigate numerosity while keeping heterogeneity constant (i.e., it is usually infeasible to present identical phonological variation across single-talker and multiple-talker exposure conditions). Meanwhile, even if the number of exposure talkers is stable (thus controlling for numerosity), probing heterogeneity accounts also brings its own challenges. L2-accented and L1-accented sentences potentially differ on dozens of phonological contrasts (e.g., word-final stop consonants, tense/lax vowels, etc.; Wang et al., 2025), with each contrast being highly multidimensional (e.g., word-final stop consonants can be cued by vowel duration, burst duration, and/or closure duration; Xie & Myers, 2017). In attempting to adjust the amount of phonological variation, it becomes difficult to determine which contrasts and which dimensions to manipulate and how much to alter them to achieve a robust effect. Although disentangling numerosity and heterogeneity accounts could be simplified by focusing on single contrasts (e.g., /b/ vs. /p/; Clayards et al., 2008), this would sacrifice the ecological validity of the sentence-length stimuli used in prior work (Bradlow & Bent, 2008; Xie et al., 2021).

For researchers examining L2-accent adaptation through more naturalistic experimental paradigms (e.g., speech-transcription-in-noise), resolving the numerosity-heterogeneity confound is an inherently difficult task without a straightforward solution. If the confound cannot be entirely controlled, one might ask the following as a starting point: Is there a way of adjusting one factor (numerosity or heterogeneity) while holding the other as constant as possible? This study introduces *apparent talker variability* (the simulation of multiple-talker exposure) as a potential method of elevating numerosity while minimizing changes in heterogeneity.

### 1.3 Leveraging apparent talker variability to increase numerosity and minimize heterogeneity

Talker variability can be simulated via the “Change Gender” function (Boersma & Weenink, 2023), a Praat algorithm that “uses pitch-synchronous overlap-and-add (PSOLA) to allow independent manipulation of formant frequencies, median F0, F0 variation, and signal duration while maintaining all of the other acoustic parameters” (Merritt & Bent, 2022, p. 487). Passing stimuli through the Change Gender function can successfully alter both the apparent gender and identity of a talker, according to anecdotal evidence (e.g., sufficiently decreasing the formant frequencies and F0 of a female speaker can make listeners think that they are hearing a novel “male” talker; Luthra et al., 2021). Thus, even if all stimuli originate from one speaker in reality, the *illusion* of multiple talkers can be created with most of the acoustic properties remaining constant (i.e., an increase in numerosity with only minor changes in heterogeneity).

Although the presentation of multiple apparent talkers would increase variability in terms of F0 and formant frequencies, this caveat is tempered by a useful feature of the Change Gender function: the adjustment of “the spectral envelope of speech sounds up/down according to *uniform scaling*” (Barreda & Silbert, 2023, Section 13.2.2; emphasis added). Recent work in the normalization literature demonstrates that uniform scaling does not affect vowel identification—when all formants of a particular vowel are scaled up or down by the same ratio, the stimulus is still heard as the exact same vowel with the same quality (Barreda, 2021). According to Barreda (2020), listeners only associate shifts in uniform scaling with anatomical and/or social differences (e.g., vocal tract length, speaker size, and speaker gender), not with phonological differences. Phonological differences are only perceived when nonuniform scaling occurs (e.g., when the proportional increase in the first formant (F1) is greater than the other formants, a vowel should be judged as relatively lowered; Barreda & Nearey, 2012). In short, even if *acoustic* variability in F0 and formant frequencies is heightened, this type of variation should be largely “removed by the ‘normalizing ear’ of the listener” (Barreda, 2021, p. 29).

Despite the maintenance of *most* phonological features, certain increases in variability are likely unavoidable. For instance, after running stimuli from a female talker through the Change Gender function to generate a “novel male” talker, Cummings and Theodore (2022) reported inadvertent alterations in fricative acoustics:

. . . though the Change Gender parameters do not suggest nor explicitly allow control over changes in voiceless energy [of /s/ and /ʃ/], this function does introduce a scaling of center of gravity in line with the change in fundamental frequency. Specifically, center of gravity for [the “novel male”] tokens is shifted towards lower frequencies compared with [the original female] tokens. (p. 2340)

Employing the Change Gender function therefore does not entirely eliminate the numerosity-heterogeneity confound—rather, echoing the stated goal at the end of Section 1.2, the proposed method increases numerosity while keeping changes in heterogeneity to a minimum.

The Change Gender function allows for two types of conditions to be compared—single-talker exposure (where all stimuli come from one talker and are unmanipulated) and multiple apparent talker exposure (where all stimuli come from one talker, but some are manipulated to alter apparent identity). A numerosity account predicts that multiple apparent talker exposure should enhance generalization. By contrast, a null effect of apparent talker variability seems more likely for a heterogeneity account, given the minimized change in phonological variation across exposure conditions.

### 1.4 Potential effects of speaking style and acoustic similarity on generalization

If numerosity and heterogeneity are both plausible accounts (Raviv et al., 2022), why do Xie et al. (2021) observe equivalent effects of multiple-talker and single-talker exposure on generalization? As a further quandary, any facilitatory effects of numerosity and heterogeneity likely interact with similarity. A *similarity account* predicts generalization to occur when at least one exposure talker is sufficiently similar phonologically to the novel talker (e.g., an alignment in the use of vowel duration, burst duration, and closure duration when distinguishing word-final stop consonants; Xie & Myers, 2017). Merely increasing the number of exposure talkers thus does not guarantee a boost in generalization if the additional talkers are not similar enough to the novel talker.

Akin to numerosity and heterogeneity, similarity accounts can be difficult to evaluate, especially when working with sentence-length stimuli. As discussed in Section 1.2, numerous phonological contrasts can be heard within a set of sentences, and it becomes challenging to determine which features are important for assessing between-talker similarity. Another issue is that individual differences in cue weighting are well-documented (Clayards, 2018; Yu & Zellou, 2019), meaning that even for the same phonological contrast, listeners might diverge in their perceptual judgments of similarity. For example, if the word-final stop consonants of an exposure and novel talker are only alike in burst duration, but not in closure or vowel duration, then generalization might be facilitated for listeners attuned to burst duration, but blocked for those who weight closure or vowel duration more heavily. Defining “similarity” is ultimately not straightforward, and adaptation studies with sentence-length stimuli must often resort to exploratory, post hoc assessments of similarity for a limited subset of features (Bradlow et al., 2023; Sidaras et al., 2009).

One way of alleviating these issues is to examine *speaking style*. Speaking style is a ubiquitous type of within-talker variation, referring to systematic shifts in production based on the social context and/or interlocutor (Aoki & Zellou, 2023a, 2023b; Smiljanić, 2021; Vonessen et al., 2024). Both L1- and L2-accented speakers adjust their productions based on listener identity—for instance, when asked to imagine talking to someone who is hard-of-hearing, speakers make exaggerated acoustic-phonetic modifications (e.g., expanded vowels, greater F0 variation, slower speaking rate) relative to their default, “casual” mode of speaking (Kato & Baese-Berk, 2022; Scarborough & Zellou, 2013). Perceptual effects of speaking style are usually consistent, with hyper-articulated styles like hard-of-hearing-directed speech tending to enhance intelligibility in noise compared with casual speech (Aoki & Zellou, 2024; Picheny et al., 1985).

Introducing speaking style into L2-accent adaptation experiments does not completely eliminate the aforementioned difficulties about measuring similarity. Speaking styles are highly multidimensional acoustically and phonologically (Smiljanić, 2021), and perceptual effects can vary across listeners (Ferguson & Kewley-Port, 2002). Nevertheless, when employing sentence-length stimuli, speaking style proffers an a priori hypothesis about similarity effects without needing to rely on post hoc analyses. In particular, a similarity account predicts that generalization should be facilitated if the styles of the exposure and novel talkers are congruent.

### 1.5 This study

This study employed the speech-transcription-in-noise paradigm (Aoki & Zellou, 2023c; Bradlow & Bent, 2008) to investigate how apparent talker variability and speaking style impact cross-talker generalization of L2-accented speech. Listeners were either assigned to a critical condition (an exposure phase followed by a test phase) or to a control condition (no exposure, as in Bradlow et al. (2023)). The critical conditions presented one Mandarin-accented English talker in exposure and one novel talker from the same language background at test.

The experiment had a 5 × 2 between-participant design. There were five possible exposure conditions: (a) no exposure; (b) a single talker producing casual speech; (c) a single talker producing hard-of-hearing-directed speech; (d) multiple apparent talkers producing casual speech; and (e) multiple apparent talkers producing hard-of-hearing-directed speech. There were also two test conditions: (a) a single talker producing casual speech and (b) a single talker producing hard-of-hearing-directed speech.

Importantly, the exposure stimuli were originally produced by a single talker across all critical conditions—the number of *apparent* talkers was manipulated by uniformly scaling F0 and formant frequencies. Simulations of multiple-talker exposure in the speech-transcription-in-noise paradigm represents a first pass at simultaneously: (a) reducing the numerosity-heterogeneity confound typifying perceptual studies on talker variability (Raviv et al., 2022) and (b) employing a naturalistic experimental task.

The present experiment addresses two broad questions, which are each addressed in separate subsections. Question 2 reflects the principal aim of this study, while Question 1 is a critical precursor that must be answered before proceeding to Question 2.

#### 1.5.1 Question 1: How do speaking style and apparent talker variability influence exposure phase intelligibility?

Although cross-talker generalization is of primary interest (i.e., differences in test phase accuracy across conditions), it is essential to affirm that the exposure phase manipulations are perceived as intended. More specifically, one must (a) establish that casual and hard-of-hearing-directed speech differ perceptually and (b) verify that the listeners who hear multiple apparent talkers are actually perceiving different talkers.

A benefit of the current experimental design is that both of these concerns can be addressed by analyzing the exposure phase results. First, exposure phase transcription accuracy is expected to be higher for hard-of-hearing-directed speech than for casual speech (Aoki & Zellou, 2024; Picheny et al., 1985). Second, if the apparent talker manipulation is effective, then it should replicate prior work on the perception of (veridical) talker variability. In other words, exposure phase intelligibility should be greater when listening to a single talker than to multiple apparent talkers (Bent & Holt, 2013; Mullennix et al., 1989).

Note that the predicted effect of apparent talker variability differs between the exposure and test phases. As noted by Raviv et al. (2022), “greater variability may initially hinder learning [e.g., lower transcription accuracy in exposure], but typically leads to an improved ability to generalize learning to new contexts [e.g., greater transcription accuracy at test for a novel talker]” (p. 463). Since the current work is principally concerned with generalization, an explanation of why talker variability might “initially hinder learning” in exposure is outside the scope of this study—for a detailed review, see Luthra (2024).

#### 1.5.2 Question 2: How do speaking style and apparent talker variability influence cross-talker generalization?

Following Bradlow et al. (2023), cross-talker generalization is defined as increased test phase transcription accuracy compared with the no-exposure control condition. All critical conditions with exposure are expected to enhance test phase accuracy, given recent evidence that single-talker exposure can be sufficient for generalization (Xie et al., 2021). However, different theoretical accounts make distinct predictions about which (or whether) specific conditions should show an additional generalization enhancement.

A *similarity account* predicts that generalization should be facilitated when there is an alignment in speaking style between the exposure and test phases. In other words, listeners exposed to casual speech should only show an extra boost in test phase accuracy when tested on casual speech. Meanwhile, participants presented with hard-of-hearing-directed speech in exposure should only benefit more perceptually when tested on hard-of-hearing-directed speech.

Numerosity and heterogeneity accounts diverge in their predictions about the effect of apparent talker variability. If greater phonological variability is necessary for enhanced generalization, as claimed by a stringent version of a *heterogeneity account*, then apparent talker variability is unlikely to impact test phase accuracy. By contrast, a *numerosity account* states that increasing the number of perceived talkers should enhance generalization, regardless of phonological variability—in this case, exposure to multiple apparent talkers is expected to augment generalization beyond exposure to a single talker.

## 2 Method

### 2.1 Stimuli

The speakers were four undergraduate international students from China who were recruited from the University of California (UC) Davis Psychology participant pool and compensated with course credit.^2^ All talkers were 20- to 22-year-old Asian men who had been studying English for 7–15 years, reported that their first and strongest language was Mandarin, and did not self-report knowledge of any other language besides Mandarin and English.

The recording procedure was preapproved by the UC Davis Institutional Review Board (IRB) (reference number: 1328085-2) and was identical to Aoki and Zellou (2024). Participants were first seated in front of a computer within a sound-attenuated booth. After giving informed consent, speakers completed a three-block sentence production task and a demographic questionnaire. Each block was preceded by a written prompt designed to elicit one of three speaking styles to an imagined interlocutor: hard-of-hearing-directed speech, non-native-directed speech, or casual speech. Within each block, speakers produced the same 78 low-predictability sentences, all of which ended in a keyword (e.g., “Mr. Black knew about the *pad*”; Kalikow et al., 1977).^3^ The entire study was conducted through a self-paced Qualtrics survey, and recordings were made with a Shure WH20XLR head-mounted microphone at a digital sampling rate of 44.1-kHz. Details about block and sentence counterbalancing, the wording of the speaking style prompts, and other aspects of the recording procedure can be found in Section 2.1.3 of Aoki and Zellou (2024).

As noted in Section 1.5.1, an important preliminary step is to ensure that the speaking styles differ perceptually (in this case, that there is a robust intelligibility difference). Although both hard-of-hearing-directed and non-native-directed speech are ostensibly intended to facilitate comprehension (Piazza et al., 2022; Picheny et al., 1986), recent work suggests that only the former is actually effective at enhancing transcription accuracy in noise (Aoki & Zellou, 2024). Therefore, to maximize the intelligibility gap across conditions, the present study focused on a binary comparison between hard-of-hearing-directed speech and casual speech. Future research should explore how non-native-directed speech and other speaking styles might influence cross-talker generalization.

For each of the four male speakers, two apparently female talkers were created using the “Change gender . . .” function in Praat. Both F0 median and formant frequencies of the original stimuli were manipulated, given evidence that adjusting only F0 or only formants is insufficient for shifting apparent gender judgments (Hillenbrand & Clark, 2009). The specific parameter adjustments were based on the “h95” data set in the *phonTools* R package, which contains “[f]ormant frequency, F0 and duration information for vowels collected from 139 speakers in the Hillenbrand et al. (1995) data” (Barreda, 2015, p. 17). For the first set of apparently different talkers, F0 median was set to 220 Hz (the average F0 of the adult female speakers in h95), while the formant shift ratio equaled 1.15 (roughly the average scale factor between the adult female and adult male speakers in h95 across the first three formants). The second set of apparently different talkers was generated by setting the F0 median and formant shift ratio to 259 Hz and 1.23, respectively, matching the female talker in h95 with the highest F0. In summary, there were three apparent talkers per speaker (Original Male, Apparent Female 1, Apparent Female 2) and 1,872 total stimuli (4 talkers * 2 styles * 78 sentences * 3 apparent talkers).

To prepare the sentences for the speech-transcription-in-noise experiment, all stimuli were set to a presentation level of 65 dB SPL using Praat. Speech-shaped noise was developed from the long-term spectrum of the concatenation of all sentences (Winn, 2019). Following Bradlow and Bent (2008), stimuli were mixed with noise at a +5 dB signal-to-noise ratio (McCloy, 2015). Noise commenced 500 milliseconds before sentence onset and ended 500 milliseconds after sentence offset.

### 2.2 Acoustic analysis

Although this study is focused on speech perception, an acoustic analysis was conducted to confirm that the hard-of-hearing-directed stimuli are hyper-articulated, in alignment with prior work (Aoki & Zellou, 2024; Picheny et al., 1986). Hyper-articulation is often characterized by simultaneous adjustments on many acoustic-phonetic parameters (e.g., speaking rate, F0, vowel space area, etc.; Smiljanić & Bradlow, 2009), and it can be challenging to determine which specific cues are responsible for intelligibility enhancements (Kato & Baese-Berk, 2024). The present analysis is therefore limited in scope.

Two features were examined, both of which are modulated by L1-Mandarin speakers in English: speaking rate (Kato & Baese-Berk, 2024) and burst duration of word-final voiceless stop consonants (Xie & Myers, 2017). For all sentences, speaking rate was calculated with a custom-made Praat script and defined as the number of syllables divided by sentence duration (Cohn et al., 2021). Burst duration was only examined for sentence-final keywords that ended in a voiceless stop consonant (e.g., /p/ in “rope”) and that were released in both styles (160 total tokens across all speakers). If speakers are hyper-articulating relative to casual speech (Uchanski, 2005), then hard-of-hearing-directed speech should exhibit a slower speaking rate (fewer syllables per second) and a longer burst duration (i.e., signaling an exaggerated contrast relative to voiced stop consonants, which tend to have shorter burst durations).

Table 1 shows the results of paired t-tests comparing hard-of-hearing-directed and casual speech for each speaker on each acoustic cue. Despite some variation in effect size, all speakers were acoustically similar to each other, producing a significantly slower speaking rate and longer burst duration in hard-of-hearing-directed speech than casual speech. These results verify that the hard-of-hearing-directed stimuli are hyper-articulated and thus consistent with previous research.

**Table 1.** Results of the Acoustic Analysis for Each Speaker.

Speaker	Burst duration (ms)					Speaking rate (syll/s)				
	Mean Diff	95% CI	*t*-value	*df*	*p*-value	Mean Diff	95% CI	*t*-value	*df*	*p*-value
BF	35.66	[10.91, 60.41]	3.21	10	0.009**	−1.83	[–1.97, –1.68]	−25.12	77	<0.001***
BX	50.89	[27.59, 74.20]	4.56	20	<0.001***	−1.53	[–1.70, –1.37]	−18.99	77	<0.001***
DW	21.48	[7.52, 35.44]	3.18	23	0.004**	−0.90	[–1.01, –0.79]	−15.94	77	<0.001***
TW	16.42	[1.28, 31.56]	2.24	23	0.03*	−1.01	[–1.10, –0.91]	−21.20	77	<0.001***

*Note*. The “Mean Diff” column subtracts the average value for hard-of-hearing-directed speech by the average value for casual speech. The full output of paired t-tests are reported: 95% confidence intervals (CI), *t*-values, degrees of freedom (*df*), and *p*-values. Significance annotations reflect meaningful differences between styles.

* = *p* < .05; ** = *p* < .01; *** = *p* < .001.

### 2.3 Participants

The current experiment has a fully between-participant design (see Section 2.4), similar to many prior studies on L2-accent adaptation (e.g., Baese-Berk et al., 2013; Bradlow & Bent, 2008; Xie et al., 2021). Given the lack of within-participant variables, a sufficiently large sample size is especially critical to ensure that any differences between conditions are not merely due to listener idiosyncrasies (Brysbaert & Stevens, 2018). The target sample size was thus 800 participants (*n* = 80 across 10 conditions; see Section 2.4), matching the number of listeners per group in Xie et al. (2021). After running various simulations, Xie et al. (2021) achieved 45.9%–96.4% power with 80 listeners in each condition—this was deemed acceptable, since power was considerably higher than the 21%–38.4% range of the original Bradlow and Bent (2008) experiment.

Participants were recruited from Prolific and paid approximately US$9 per hour (US$1.40 if placed in the shorter condition without an exposure phase and US$2.50 if placed in any other group; see Section 2.4 for details). All 909 participants gave informed consent before the study, which was approved by the UC Davis IRB (reference number: 1328085-2). The demographic filters on Prolific were used to narrow down the participant pool to individuals who were born and living in the United States, between 18 and 35 years old, and whose self-reported first language was English (these specific criteria were selected to align with prior speech-transcription-in-noise studies; for example, Experiment 3 of McLaughlin, 2022).

Certain participants (*n* = 94) were omitted from the analysis (the 10.3% exclusion rate is comparable with Xie et al. (2021), who reported 6.7% and 15.6% exclusion rates for their first and second experiments, respectively). Listeners were removed based on the following self-reported criteria: (a) audio, technical, and/or hearing difficulties (*n* = 31); (b) English not being their dominant language (*n* = 7); (c) childhood and/or daily exposure to any Chinese language or dialect (Mandarin, Cantonese, Mien, Hokkien; *n* = 49); (d) working proficiency or fluency in Mandarin (*n* = 3); (e) being older than 35 years old (i.e., a mismatch from their official profile on Prolific; *n* = 3); and (f) writing nonwords or “don’t know” on the majority of trials in the test phase of the transcription task (*n* = 1).

Slightly exceeding the original sample size target, the final analysis included data from 815 participants (445 women, 340 men, 30 nonbinary; mean age = 28.01 years, SD = 4.54; self-reported ethnicity: Asian = 70, Black = 107, Latino = 49, Mixed = 124, Native American or Alaska Native = 4, White = 461).

### 2.4 Procedure

#### 2.4.1 Pre-experiment

The entire study was completed through an online, self-paced Qualtrics survey. After providing informed consent, participants received instructions to wear headphones and to move to a quiet area with little background noise (explicit headphone and background noise checks were not built into the survey).

A short procedure ensued that functioned as both an informal sound calibration and an informal attention check (this task was identical to that reported in Section 2.1 of Zellou et al. (2023)). Sound calibration was the primary goal—participants listened to a single sentence [“She asked about the host”] and were asked to adjust the volume of their computer to a comfortable level, only if they felt it was necessary. They were asked not to alter their volume for the remainder of the experiment.

To ensure that participants had listened to the sentence, a mandatory, forced-choice question was included as an attention check. Listeners were asked to identify the sentence from among three options (“She asked about the host,” “She asked about the toast,” “She asked about the “coast”). Proceeding to the next phase of the Qualtrics survey was blocked until the correct answer was provided. The question could be taken an unlimited number of times and did not function as a screener. All participants eventually gave the correct response and were not removed if they initially answered the question incorrectly.

The stimulus was amplitude-normalized to 60 dB SPL (a similar amplitude as the actual experimental stimuli) and was produced by a male, text-to-speech voice (“Matthew” from Amazon Polly; Amazon Web Services, n.d.). There was no specific reason to use this particular voice, other than following past work (e.g., Zellou et al., 2023). The stimulus could be played up to five times. Replaying the stimulus was not required, but included as an option just in case participants wanted to adjust their volume level and/or had answered the forced-choice question incorrectly and wanted to hear the sentence again.

After successfully answering the multiple-choice question, listeners then completed a perceptual experiment and filled out a demographic questionnaire.

#### 2.4.2 Experiment

The experiment was a speech-transcription-in-noise task. The 78 low-predictability sentences described in Section 2.1 were evenly divided across a 39-trial exposure phase and a 39-trial test phase, with each sentence only ever appearing in one of the phases (e.g., “Mr. Black knew about the pad” was always heard in the exposure phase, never in the test phase). Each trial auditorily presented a single sentence masked by noise, and listeners were asked to type the final word into a text box. All sentences were presented in a pseudo-randomized order and only played once with no feedback provided.

Participants were randomly assigned to one of five exposure conditions and one of two test conditions (5 exposure × 2 test = 10 possible conditions overall). In terms of exposure, listeners either heard: (a) no exposure (the same control in Bradlow et al. (2023)); (b) a single talker producing casual speech; (c) a single talker producing hard-of-hearing-directed speech; (d) multiple apparent talkers producing casual speech; (e) multiple apparent talkers producing hard-of-hearing-directed speech. The non-control exposure conditions are crossed in a 2 × 2 design between Exposure Style (casual, hard-of-hearing-directed) and Apparent Exposure Talker Number (single, multiple).

Importantly, the exposure stimuli always originated from a single Mandarin-accented English talker—listeners merely differed in the number of *apparent* talkers heard in exposure. Whereas the single talker conditions just presented the veridical (male) stimuli, the multiple apparent talker conditions presented three seemingly different talkers created through the “Change Gender” Praat function (see Section 2.1 for details). To reinforce the experimental manipulation, listeners who heard multiple apparent talkers were explicitly told in the exposure phase instructions that they would be hearing “three speakers.” Sentence content and apparent talker assignment were evenly counterbalanced.

All participants completed a test phase, in which a single (novel) Mandarin-accented English talker produced either casual or hard-of-hearing-directed speech. Only the original productions were presented, so apparent talker variability was not manipulated. Based on Xie et al. (2021), each of the four speakers were selected as the exposure and test talkers equally often.

### 2.5 Data processing and statistical analysis

The exposure and test phase responses were cleaned by removing all spaces and punctuation, converting all characters to lowercase, and deleting any nonfinal keywords (e.g., when an entire sentence was transcribed instead of just the final keyword). Accuracy was then coded binomially as either correct (=1) or incorrect (=0). Similar to prior work (Aoki & Zellou, 2023c; Bent & Bradlow, 2003), a strict standard was employed, with responses only counted as correct if they contained all and only the appropriate affixes (e.g., if “cake” was the keyword, “cakes” was given a 0).^4^ However, both obvious spelling errors (e.g., “sleaves” for “sleeves”) and homophones (e.g., “greece” for “grease”) were considered correct.

Statistical analysis was conducted with Bayesian mixed-effects logistic regression in R (R Core Team, 2021) through the *brms* package (Bürkner, 2017) and *Stan* (Stan Development Team, 2023). Several analyses were conducted, with each analysis answering a distinct question (for clarity, the syntax for each model is described in Section 3). The models all contained between-participant fixed effects and prior distributions that aligned with prior work (specifically, the logistic regression model described in Chapter 10.5.2 of Barreda and Silbert (2023)). Effects are interpreted as meaningful if 95% credible intervals do not contain zero.

#### 2.5.1 Motivating the random effects structure of the statistical models

Researchers are always faced with choices (“degrees of freedom”) in data analysis (Simmons et al., 2011). One major area of uncertainty and divergence among psycholinguists (and others relying heavily on experiments with repeated-measures designs) lies in the specification of random effects (Coretta et al., 2023; Meteyard & Davies, 2020). In particular, there are two predominant philosophies regarding the modeling of random slopes: maximal and data-driven. The maximal approach advocates for “add[ing] as many random slopes as possible,” while the data-driven perspective recommends only “add[ing] random slopes that significantly improve the model” (Sonderegger, 2023, p. 264). Deciding on the appropriate model structure is made challenging by the following trade-off—inserting random slope terms tends to decrease the Type I error (false positive) rate (Barr et al., 2013), while also potentially increasing the Type II error (false negative) rate (Matuschek et al., 2017).

For the current experiment, 11 models were fitted for the analysis (one for the exposure phase, two for just the critical conditions of the test phase, and eight for comparing the control conditions to the critical conditions of the test phase; see Section 3 for details). All models included by-listener, by-talker, and by-sentence random intercepts by default (the inclusion of all possible random intercepts is not controversial; Sonderegger, 2023, p. 265). However, due to concerns about computational feasibility, a frequentist, forward-selection, data-driven procedure was employed to evaluate the effect of random slopes on model fit (note that only by-talker and by-sentence random slopes were considered—by-listener random slopes would not be appropriate due to the fully between-participant design of the current experiment; Barr et al., 2013).

Using the (frequentist) *lme4* and *lmerTest* packages (Bates et al., 2015; Kuznetsova et al., 2017), all random slopes were added one at a time to all 11 models, with likelihood-ratio tests (LRT) through *anova()* used to determine whether the model had been “improved” (defined as an LRT with *p* < .2, a conservative threshold recommended by Matuschek et al., 2017). If an LRT reported *p* < .2, the slope was retained and the next slope was added to this new model and tested. Where two slopes were retained, an interaction was tested. If an LRT reported *p* > .2, then this random slope and any interactions were not included in the model and the next slope was tested.

The results of LRTs differed between the exposure and test phases. For all 10 test phase models, a nonmaximal random effects structure was sufficient (random-intercept-only models were sufficient for nine of 10). However, the maximal random effects structure was helpful in improving the modeling of the exposure phase (*p* = .07 for the final LRT comparing the maximal model to a near-maximal model with one random slope removed).

There are two reasons why Section 3 (“Results”) solely reports the output of random intercept only models. First, since the exposure phase model has an identical fixed effect structure as one of the test phase models (see equation 1), consistency and writing clarity are enhanced if both models also have an identical random effects structure. Second, there was no difference in interpretation between the random-intercept-only model and the maximal random slopes model for the exposure phase (both outputs show two statistically significant main effects [*p* < .05] with no significant interaction [*p* > .39]). The same held true for the tenth test phase model (both the random-intercept-only and the nonmaximal random slopes model revealed a statistically significant main effect [*p* < .05], a trending effect [*p* < .10], and no other significant effects [*p* > .19]).

For transparency, the models with random slopes and the R script used to compare the models can be found through the “Data Availability” statement.

## 3 Results

### 3.1 Question 1: How do speaking style and apparent talker variability influence exposure phase intelligibility?

The first analysis examined effects of speaking style and apparent talker variability on exposure phase transcription accuracy. The statistical model (depicted in equation [1] with R syntax) was fitted with sum-coded fixed effects of Exposure Style (casual, hard-of-hearing-directed) and Apparent Exposure Talker Number (single, multiple), as well as their interaction. By-listener, by-talker, and by-sentence random intercepts were included.

(1) $$\begin{array}{l} \mathrm{Accuracy}\;\sim \;\mathrm{Exposure}\;\mathrm{Style}\;*\;\mathrm{Apparent}\;\mathrm{Exposure}\;\mathrm{Talker}\;\mathrm{Number}\;+\\ \;\left(1\;|\;\mathrm{Listener}\right)\;+\;\left(1\;|\;\mathrm{Talker}\right)\;+\;\left(1\;|\;\mathrm{Sentence}\right) \end{array}$$

**Figure 1.**
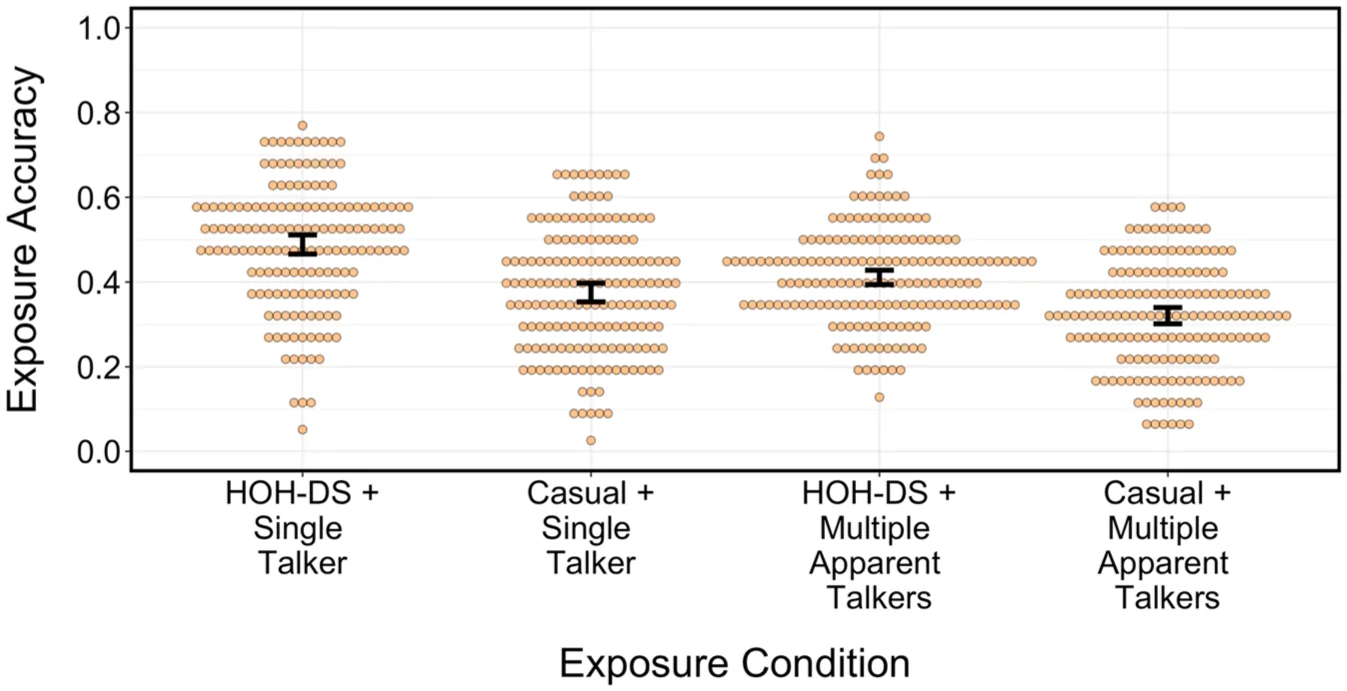
(Color online). Exposure phase transcription accuracy across conditions. Individual points reflect by-participant mean performance. Error bars are 95% confidence intervals bootstrapped over by-participant means.

Figure 1 visualizes the exposure phase results (note that the abbreviation “HOH-DS” stands for “hard-of-hearing-directed speech” in all figures). A meaningful main effect of Exposure Style was revealed (β = −0.29, *SE* = 0.02, 95% highest density interval [HDI] = [−0.33, −0.24]), indicating that hard-of-hearing-directed speech was transcribed more accurately than casual speech. There was also a main effect of Apparent Exposure Talker Number (β = 0.19, *SE* = 0.02, 95% HDI = [0.14, 0.23]), highlighting a comprehension detriment for listeners who heard multiple apparent talkers. No interaction was observed (β = −0.02, *SE* = 0.02, 95% HDI = [−0.07, 0.03]), which implies that the effect of Exposure Style did not differ meaningfully based on the number of apparent exposure talkers.

These findings are highly consistent with prior work. First, the hyper-articulated, acoustic-phonetic modifications in the hard-of-hearing-directed productions (see Section 2.2) are successful at enhancing intelligibility, aligning with the literature on speaking style perception (Aoki & Zellou, 2024; Picheny et al., 1986). Moreover, akin to past studies on (veridical) talker variability (Bent & Holt, 2013; Mullennix et al., 1989), listening to multiple apparent talkers hinders perception in initial exposure—this suggests that the simulation of talker variability is effective at inducing the perception of multiple talkers.

### 3.2 Question 2: How do speaking style and apparent talker variability influence cross-talker generalization?

#### 3.2.1 Phase 1: Did generalization occur at all? A comparison between the control and critical conditions

Following Cummings and Theodore (2023), the test phase analysis was divided into two stages. The initial stage assessed whether prior exposure facilitated generalization at all. This was evaluated with eight separate models, which compared each of the eight critical conditions to a no-exposure control condition. The comparisons were always within speaking styles (e.g., listeners tested on casual speech were only compared with participants who were similarly tested on casual speech). Each model, whose syntax is shown in equation (2), contained a treatment-coded fixed effect of Condition (two levels, reference = no-exposure control) and the same random effects structure as equation (1).

(2) Accuracy~Condition+(1|Listener)+(1|Talker)+(1|Sentence)

**Figure 2.**
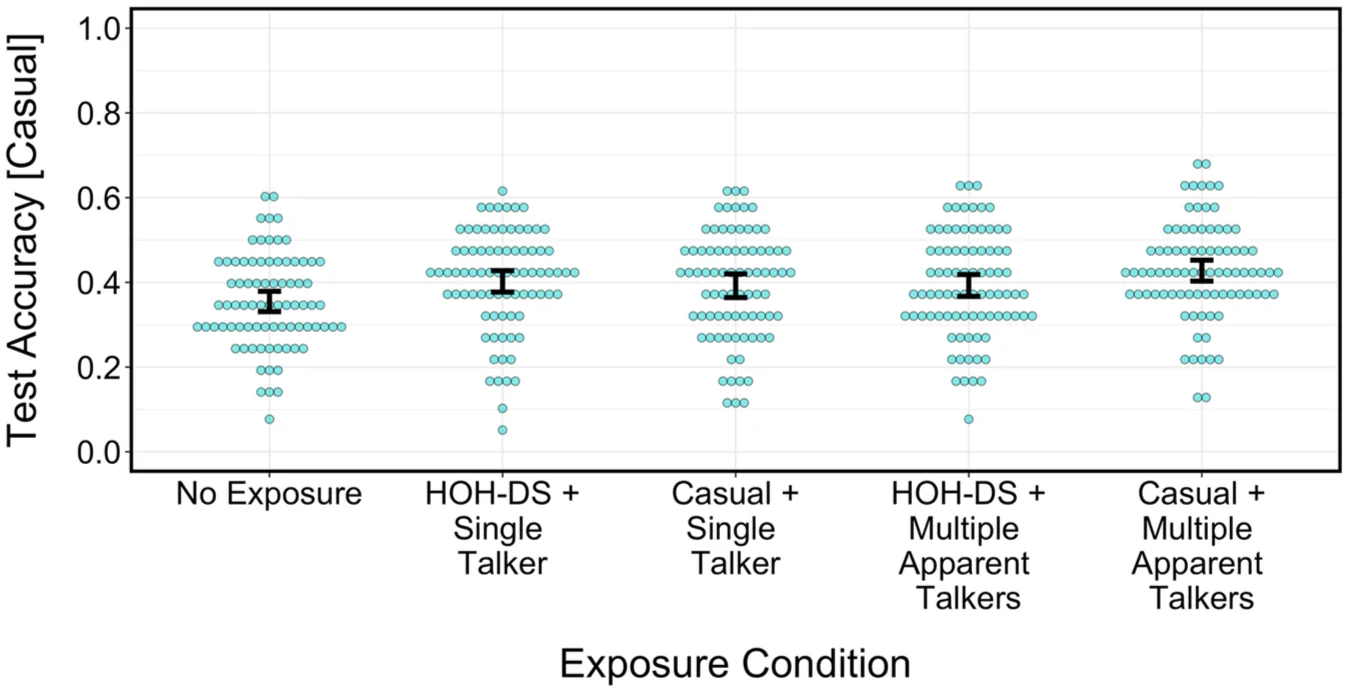
(Color online). Test phase transcription accuracy across conditions, among participants tested on casual speech. Individual points reflect by-participant mean performance. Error bars are 95% confidence intervals bootstrapped over by-participant means.

**Figure 3.**
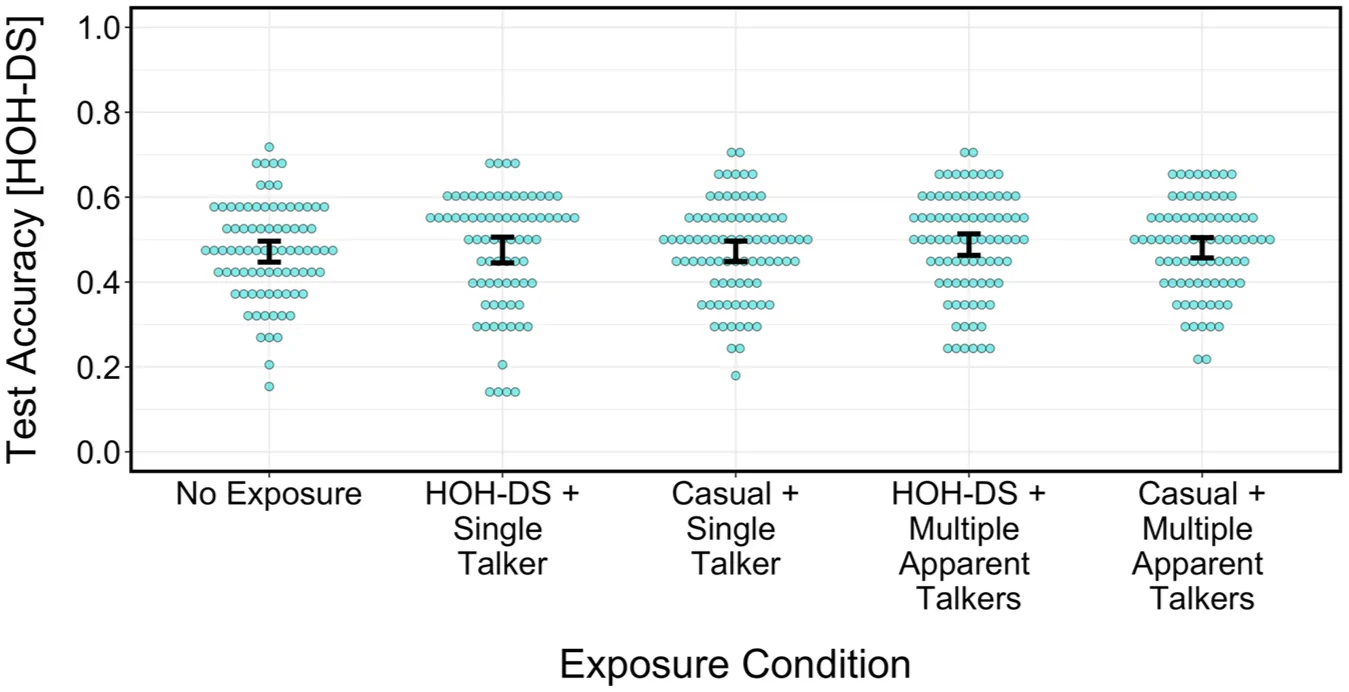
(Color online). Average test phase transcription accuracy across conditions, among participants tested on hard-of-hearing-directed speech. Individual points reflect by-participant mean performance. Error bars are 95% confidence intervals bootstrapped over by-participant means.

The test phase results are divided into two images for clarity, with Figures 2 and 3 showing performance on casual speech and hard-of-hearing-directed speech, respectively. Among listeners tested on casual speech, all four conditions with exposure facilitated cross-talker generalization. More specifically, compared with the no-exposure control, test phase accuracy was greater among listeners exposed to: (a) hard-of-hearing-directed speech and a single apparent talker (β = 0.28, *SE* = 0.08, 95% HDI = [0.12, 0.45]); (b) casual speech and a single apparent talker (β = 0.22, *SE* = 0.09, 95% HDI = [0.03, 0.40]); (c) hard-of-hearing-directed speech and multiple apparent talkers (β = 0.20, *SE* = 0.09, 95% HDI = [0.03, 0.38]); (d) casual speech and multiple apparent talkers (β = 0.41, *SE* = 0.08, 95% HDI = [0.25, 0.57]).

By contrast, no generalization took place for listeners tested on hard-of-hearing-directed speech. Relative to the no-exposure control, no meaningful difference in test phase performance emerged for any condition: (a) hard-of-hearing-directed speech and a single apparent talker (β = 0.03, *SE* = 0.09, 95% HDI = [−0.14, 0.20]); (b) casual speech and a single apparent talker (β = 0.02, *SE* = 0.07, 95% HDI = [−0.13, 0.17]); (c) hard-of-hearing-directed speech and multiple apparent talkers (β = 0.10, *SE* = 0.08, 95% HDI = [−0.07, 0.26]); (d) casual speech and multiple apparent talkers (β = 0.04, *SE* = 0.08, 95% HDI = [−0.11, 0.19]).

In summary, there was evidence of cross-talker generalization, but only for listeners tested on casual speech (not for listeners tested on hard-of-hearing-directed speech).

#### 3.2.2 Phase 2: Investigating the relative degree of generalization across critical conditions

The second stage of the test phase analysis investigated the relative degree of generalization across the critical conditions. Since no generalization took place among listeners tested on hard-of-hearing-directed speech, the initial statistical model in this section only includes results for participants tested on casual speech (i.e., the four right-most conditions in Figure 2). The model formula was identical to equation (1).

There were no main effects of Exposure Style (β = 0.03, *SE* = 0.03, 95% HDI = [−0.03, 0.10]) or Apparent Exposure Talker Number (β = −0.03, *SE* = 0.03, 95% HDI = [−0.09, 0.03]). However, a marginally meaningful interaction emerged (β = −0.06, *SE* = 0.03, 95% HDI = [−0.13, −0.002]). The interaction term was interpreted via the *hypothesis* function from the *brms* package (Barreda & Silbert, 2023). This function is useful, since it allows researchers to investigate interactions without fitting additional post hoc models (thus avoiding loss of statistical power).

The first hypothesis revealed no effect of apparent talker variability for listeners exposed to hard-of-hearing-directed speech (β = 0.03, *SE* = 0.04, 95% HDI = [−0.05, 0.12]). By contrast, exposure to multiple apparent talkers led to a meaningful increase in test phase accuracy for listeners exposed to casual speech (β = −0.09, *SE* = 0.04, 95% HDI = [−0.18, −0.008]). In other words, among listeners tested on casual speech (Figure 2), the No Exposure condition had the lowest accuracy, the “Casual + Multiple Apparent Talkers” condition had the highest accuracy, and the other three exposure conditions had an equally intermediate accuracy.

This interaction is quite weak, and it remains unclear whether the effect would remain in an aggregated analysis that combined all critical conditions (i.e., the four right-most conditions in Figure 2 *and* the four-most conditions in Figure 3). This question was investigated through a final statistical model that added Test Style (casual, hard-of-hearing-directed speech) as a sum-coded fixed effect to equation (1), along with all possible interactions (see equation [3]). Since no generalization was observed among listeners tested on hard-of-hearing-directed speech (see Section 3.2.1), then there should be a three-way interaction in the current model—Exposure Style and Apparent Exposure Talker Number should jointly influence test phase accuracy, but only for listeners tested on casual speech.

(3) $$\begin{array}{l} \mathrm{Accuracy}\;\sim \;\mathrm{Exposure}\;\mathrm{Style}\;*\;\mathrm{Apparent}\;\mathrm{Exposure}\;\mathrm{Talker}\;\mathrm{Number}\;*\;\mathrm{Test}\;\mathrm{Style}\;\\ +\;\left(1\;|\;\mathrm{Listener}\right)\;+\;\left(1\;|\;\mathrm{Talker}\right)\;+\;\left(1\;|\;\mathrm{Sentence}\right) \end{array}$$

The full summary statistics of this final model are displayed in Table 2 for clarity. A meaningful effect of Test Style was observed (β = −0.21, *SE* = 0.02, 95% HDI = [−0.25, −0.17]), indicating that casual speech was transcribed less accurately overall than hard-of-hearing-directed speech. Only a marginal three-way interaction emerged between Exposure Style, Test Style, and Apparent Exposure Talker Number (β = −0.04, *SE* = 0.02, 95% HDI = [−0.08, 0.007]). Output from the *hypothesis* function showed: (a) marginal evidence of a two-way interaction between Exposure Style and Apparent Exposure Talker Number for listeners tested on casual speech (β = −0.06, *SE* = 0.03, 95% HDI = [−0.12, 0.0003]); (b) no evidence for such an interaction for listeners tested on hard-of-hearing-directed speech (β = 0.01, *SE* = 0.03, 95% HDI = [−0.04, 0.07]).

**Table 2.** Summary Statistics for the Final Model in Phase 2 of the Analysis.

Effect	Estimate	Est. error	l-95% CI	u-95% CI
Intercept	−0.34	0.48	−1.30	0.60
Exposure Style	0.006	0.02	−0.04	0.05
Apparent Exposure Talker Number	−0.02	0.02	−0.07	0.02
Test Style	−0.21	0.02	−0.25	−0.17
Exposure Style: Apparent Exposure Talker Number	−0.02	0.02	−0.07	0.02
Exposure Style: Test Style	0.03	0.02	−0.01	0.07
Test Style: Apparent Exposure Talker Number	−0.004	0.02	−0.05	0.04
Exposure Style: Test Style: Apparent Exposure Talker Number	−0.04	0.02	−0.08	0.007

*Note*. This model investigated the relative degree of cross-talker generalization across all critical conditions (i.e., a comparison of test phase accuracy in the four right-most conditions of Figure 2 and the four right-most conditions of Figure 3).

## 4 General discussion

A sizable literature has probed whether exposure to talker variability enhances subsequent perception of novel talkers (i.e., cross-talker generalization). These inquiries have led to mixed results. Certain studies report a facilitatory effect of multiple-talker exposure (Bradlow & Bent, 2008; Clopper & Pisoni, 2004), while others observe no difference compared with single-talker exposure (Xie et al., 2021; Xie & Myers, 2017). Even among experiments that do find a talker variability benefit, a critical confound exists (Raviv et al., 2022)—do perceptual enhancements from multiple-talker exposure stem from the greater number of talkers (*numerosity*) or greater phonological variability (*heterogeneity*)?

Leveraging a relatively naturalistic paradigm (speech-transcription-in-noise), the present experiment assessed how apparent talker variability (the simulation of multiple-talker exposure) and speaking style mediate cross-talker generalization of L2-accented speech. Even though the exposure stimuli always originated from one Mandarin-accented English talker, the illusion of “multiple” talkers was created by adjusting the F0 and formant frequencies of certain stimuli. Critically, this manipulation aimed to minimize phonological variation, thus reducing the confound present in traditional experiments on (veridical) talker variability.

There were three main findings. First, exposure to Mandarin-accented English facilitated generalization (i.e., led to enhanced comprehension of a novel talker from the same language background), but only for listeners tested on casual speech, not hard-of-hearing-directed speech. Second, exposure to multiple apparent talkers fostered greater generalization beyond single-talker exposure, but only for participants exposed to and tested on casual speech (as opposed to listeners who heard a mismatch in speaking styles across exposure and test). Third, the benefit of multiple apparent talker exposure has a fairly weak effect size.

These results are not readily explained by an extreme version of a heterogeneity account. Since differences in phonological variation were minimized between the single-talker and multiple apparent talker conditions, exposure to greater phonological variability does not seem strictly necessary for a generalization boost. Instead, the results lend credence to numerosity and similarity accounts. Increasing the number of perceived exposure talkers can be sufficient for enhancing generalization, but only if there is sufficient acoustic similarity in speaking style between the exposure and novel talkers.

The rest of the general discussion is organized as follows. Section 4.1 explores potential mechanisms of the apparent talker variability effect. Section 4.2 provides some possible explanations for why listeners tested on hard-of-hearing-directed speech unexpectedly showed no generalization. Section 4.3 discusses some methodological implications of the present work. Finally, Section 4.4 focuses on limitations of this study, highlighting avenues for future work on apparent talker variability.

### 4.1 Potential mechanisms of the apparent talker variability effect

The present study has focused on numerosity (an increase in the number of talkers) to explain the apparent talker variability effect. However, an entirely different interpretation might also be possible—what if the results of the current experiment are mediated by cognitive difficulty? Listeners exposed to casual speech and multiple apparent talkers showed the lowest transcription accuracy in the exposure phase (see Figure 1). In theory, being assigned to a more perceptually challenging task may have engendered increased attention to the exposure stimuli and thus led to greater generalization at test (Tzeng et al., 2024).

Recent work, however, provides evidence against this explanation. Employing the speech-transcription-in-noise paradigm, Bradlow et al. (2023) found that transcription accuracy for a novel L2-accented test talker was generally the lowest following exposure to a low-intelligibility Turkish-accented talker (as opposed to more intelligible Brazilian Portuguese-, Farsi-, and Spanish-accented talkers). If anything, reducing intelligibility is expected to hinder adaptation, which would not support a cognitive difficulty mechanism (see the following section for a more detailed explanation).

The ideal adapter framework more plausibly accounts for effects of apparent talker variability (Kleinschmidt & Jaeger, 2015). According to the ideal adapter framework, listeners navigate the high variability and uncertainty of the speech signal by attuning to its systematic acoustic properties (e.g., Mandarin-accented English speakers often devoice word-final stop consonants, such that “overload” is produced as “overloat”; Xie & Myers, 2017). Repeated exposure to such systematicity allows listeners to develop generative models that make predictions about future productions (e.g., a Mandarin-accented stimulus that sounds like “seat” may have been intended as “seed”). If listeners apply a generative model from a set of exposure talkers to a novel test talker (i.e., engage in cross-talker generalization) and if the talkers are acoustically similar, then test phase comprehension should increase, since listeners are more accurately predicting the test talker’s intended productions.

In this study, all listener groups tested on casual speech showed robust generalization (i.e., higher accuracy compared with the no-exposure control condition; see Figure 2). This initial finding aligns with the ideal adapter framework—since the exposure and novel talkers all came from the same language background, there was presumably sufficient acoustic similarity, which facilitated generalization (Xie et al., 2021). However, listeners exposed to casual speech and multiple apparent talkers exhibited an extra boost in test phase accuracy. This potentially occurred for two reasons: (a) there was even greater acoustic similarity across exposure and test due to speaking style alignment; (b) the *apparent* perception of multiple, acoustically similar talkers in exposure (as opposed to just a single talker) provided even greater certainty that the predictions made during exposure were truly generalizable rather than being talker-specific.

### 4.2 Why was no generalization observed among listeners tested on hard-of-hearing-directed speech?

Appealing to the ideal adapter framework only suffices for listeners tested on casual speech (i.e., when prior exposure was helpful in comprehending a novel L2-accented talker). Why was test phase accuracy the same for participants tested on hard-of-hearing-directed speech, regardless of whether or not they received prior exposure?

Perhaps there is some special property of hard-of-hearing-directed speech that blocks generalization. Hard-of-hearing-directed speech is acoustically exaggerated (see Section 2.2) and increases intelligibility in noise compared with casual speech (see Figures 2 and 3). These acoustic and perceptual enhancements might allow listeners in the no-exposure control condition to adapt more rapidly to a novel talker, rendering prior exposure unnecessary for successful adaptation. The null result in the test phase could therefore be tentatively interpreted as one of the many benefits conferred by hyper-articulated speaking styles (Smiljanić, 2021).

Looking at Figure 3, it may seem counterintuitive to treat 50% accuracy as a ceiling effect showing “successful adaptation.” This conclusion becomes more plausible, however, when the difficulty of the experiment is taken into account. Participants were not only asked to transcribe the final keyword of L2-accented sentences in noise (a demanding task in itself), but also heard each sentence just once on each trial, and were presented with challenging stimuli (low-predictability sentences such as “Miss Brown considered the *coast*”; Kalikow et al., 1977). Even experiments with significantly less taxing procedures do not necessarily lead to perfect results—for example, in the absence of background noise, participants in Kim, Chernyak, et al. (2025) show approximately 90-95% accuracy when transcribing simple sentences (e.g., “Somebody stole the money”; Bradlow, n.d.) produced by L2-accented speakers. A ceiling effect might therefore be expected at a fairly low accuracy for a more difficult task, even if listeners are exposed to hyper-articulated, hard-of-hearing-directed speech.

At the same time, the null effect observed for hard-of-hearing-directed speech might not involve any particular speaking style *per se*.^5^ Bradlow et al. (2023) point to the possible role of intelligibility on adaptation: “lexical knowledge may not be available to guide the mapping of phonetic variation to linguistically meaningful categories, and consequently, adaptation to L2 speech may be constrained for both talker-specific and talker/accent-general adaptation” (p. 1610–1611). While Bradlow et al. (2023) are careful to note the “equivocal” nature of the literature (p. 1610), low stimulus intelligibility could potentially account for both the null effects and the small effect sizes observed in this study. The average transcription accuracy across all exposure and test conditions in the present experiment is fairly low, regardless of speaking style (32-49%; see Figures 2 and 3). This is especially striking in comparison to other work—across test conditions, Table I of Bradlow et al. (2023) and Figure 3 of Xie et al. (2021) report an average accuracy of 55-89% and around 75-90%, respectively (this stark contrast likely stems from methodological differences; e.g., the use of low- vs. high-predictability sentences). The low overall intelligibility for all speaking styles in this study may have impeded adaptation, leading to both the weak effect of apparent talker variability and the lack of evidence of generalization for listeners tested on hard-of-hearing-directed speech.

In short, the null effect in this study might be driven by hard-of-hearing-directed speech itself or by low overall intelligibility. The former explanation would predict that, regardless of stimulus intelligibility, testing listeners on hard-of-hearing-directed speech should always show a null effect of exposure condition, as observed in the current work. By contrast, the latter explanation should see the null effect dissipate if listeners are presented with more easily transcribed and more intelligible stimuli.

### 4.3 On the reuse of corpora: methodological implications for research on perceptual adaptation

The discussion about speaking style in the previous section is somewhat speculative. This partially stems from a lack of precedent—although speaking style variation and perceptual adaptation are both well-researched phenomena in their own right (Bent & Baese-Berk, 2021; Smiljanić, 2021), they rarely come into contact (cf. Zhang & Samuel, 2014). The authors are not aware of any prior studies that investigate effects of speaking style on generalization of L2-accented speech.

The reuse of corpora seems to contribute to this literature gap, in addition to other issues in the field. Table 3 records all known studies (*n* = 18) that: (a) examine cross-talker generalization of L2-accented speech (i.e., comprehension of a novel L2-accented talker following an exposure or training phase); (b) employ a speech transcription or repetition task in both the exposure and test phases.^6^ The majority of studies in Table 3 (13/18) sample preexisting stimuli from one of three sources (ALLSSTAR, NUFAESD, Sidaras et al., 2009), and their repeated usage ultimately limits the scope of theoretical questions that can be asked. For example, to the authors’ knowledge, the ALLSSTAR corpus does not contain any sentence-length stimuli produced in both a hypo- and a hyper-articulated speaking style (e.g., casual and hard-of-hearing-directed speech).^7^ Thus, the ALLSSTAR corpus (and other similar resources) cannot address the questions in Section 4.2 about effects of hyper-articulation on L2-accent adaptation.

**Table 3.** A compilation of studies using a speech transcription or repetition task to examine cross-talker generalization of L2-accented speech.

Study	Corpus	Stimulus language	Listener L1	Talker L1
The Present Study	N/A	English	English	Mandarin
Bieber & Gordon-Salant (2024)	ALLSSTAR (Bradlow, n.d.) + 1 talker at University of Maryland	English	English	English, French, Hindi, Japanese, Korean, Mandarin, Portuguese, Russian, Spanish, Turkish
Nishizawa (2024)	ALLSSTAR (Bradlow, n.d.)	English	Japanese	Cantonese, English, Hindi, Indonesian, Mandarin, Spanish, Vietnamese
Tzeng et al. (2024)	Sidaras et al. (2009)	English	English	English, Spanish
Aoki & Zellou (2023c)	N/A	English	English	Mandarin
Bradlow et al. (2023)	ALLSSTAR (Bradlow, n.d.)	English	English	Farsi, Portuguese, Spanish, Turkish
McLaughlin & Van Engen (2023)	ALLSSTAR (Bradlow, n.d.)	English	English	Cantonese
Bieber & Gordon-Salant (2022)	ALLSSTAR (Bradlow, n.d.); Hoosier Database (Atagi & Bent, 2013); own recordings at University of Maryland	English	English	English, French, Hindi, Japanese, Korean, Mandarin, Portuguese, Spanish
Xie et al. (2021)	ALLSSTAR (Bradlow, n.d.)	English	English	English, Mandarin
Hau et al. (2020)	N/A	English	(Australian) English	(Australian) English, Mandarin
Alexander & Nygaard (2019)	Sidaras et al. (2009) [L1-Spanish talkers only]; own recordings at Emory University	English	English	Albanian, Bengali, Dutch, French, German, Hindi, Japanese, Korean, Mandarin, Romanian, Russian, Somali, Spanish, Turkish
Salheen et al. (2019)	N/A	English	(Libyan) Arabic	Malay
Bieber & Gordon-Salant (2017)	ALLSSTAR (Bradlow, n.d.)	English	English	Cantonese, English, Gisu, Greek, Hebrew, Japanese, Korean, Portuguese, Russian, Turkish
Tzeng et al. (2016)	Sidaras et al. (2009)	English	English	English, Spanish
Wright et al. (2015) [Experiment 2]	NUFAESD (Bent & Bradlow, 2003)	English	English	English, Mandarin
Baese-Berk et al. (2013)	NUFAESD (Bent & Bradlow, 2003)	English	English	English, Hindi, Korean, Mandarin, Romanian, Slovakian, Thai
Sidaras et al. (2009)	N/A	English	English	English, Spanish
Bradlow & Bent (2008) [Experiment 2]	NUFAESD (Bent & Bradlow, 2003)	English	English	Chinese, English, Slovakian

*Note*. There are five columns: (a) **Study** (the authors and year of publication of the article); (b) **Corpus** (in cases where the authors used prerecorded stimuli; “N/A” if the authors recorded their own stimuli); (c) **Stimulus Language** (the language of the words/sentences exposed to participants); (d) **Listener L1** (the first language of the listeners); (e) **Talker L1** (the first language of the talkers in either the exposure or test phases). Note that, in the Listener L1 and Talker L1 columns, “English” refers to “American English” unless otherwise specified.

Another issue with reusing corpora is that the recording procedure might potentially bias the results of any given experiment. For instance, according to the documentation of the ALLSSTAR corpus, speakers were taken into a sound-attenuated booth and given the following instructions: “[R]ead everything as naturally as possible. If you make a reading mistake, just pause and read the material over again as naturally as possible” (Ackerman et al., 2010, p. 20). The phrase “as naturally as possible” is somewhat vague, and some have questioned whether read speech in a controlled laboratory setting can ever truly reflect a naturalistic speaking style (Campbell-Kibler, 2009; Labov, 1972). Moreover, by asking speakers to monitor their “reading mistake[s],” the ALLSSTAR corpus instructions implicitly invite participants to pay greater attention to their speech and thus to potentially hyper-articulate more than they otherwise would (Cohn et al., 2022). The acoustic properties of the stimuli could have been affected by the instructions—it remains an open question whether adjusting the instructions would have impacted the findings of Xie et al. (2021) or other studies in Table 3 that drew stimuli from the ALLSSTAR corpus.

This discussion is not intended to imply that stimuli should never be reused, nor is it meant to critique any particular corpus or laboratory speech in and of itself (Xu, 2010). Corpora are highly convenient resources, and the authors have leveraged the ALLSSTAR corpus in some of their own pilot studies. Using the same stimuli across multiple experiments can be important for the sake of replicability (cf. Cummings & Theodore, 2023; Tzeng et al., 2021) and might even be necessary in certain extreme circumstances (e.g., when a global pandemic arises and researchers are prohibited from entering their sound booth; cf. Aoki et al., 2022; Cohn et al., 2021). The authors are not discouraging the usage of corpora in future work—rather, to achieve a richer understanding of L2-accent adaptation, studies employing corpora should be supplemented by other work using novel recordings.

### 4.4 Directions for future work

This study observes a meaningful benefit of apparent talker variability on generalization, given speaking style similarity across exposure and test phases and given the presentation of (non-hyper-articulated) casual speech at test. However, as noted in Section 4.2, the effect size is relatively small, with more research needed to make more firm conclusions. The current section delineates three concrete steps for future work.

#### 4.4.1 Addressing limitations of the speech-transcription-in-noise paradigm

This study used the speech-transcription-in-noise paradigm due to its alignment with prior seminal work on L2-accent adaptation (Bradlow & Bent, 2008) and its ecological validity, but this methodology also has at least three inherent flaws. First, the response variable (transcription accuracy) is a relatively coarse measure that only reveals whether or not adaptation occurred, but cannot shed light on the specific phonological contrasts that listeners adapted to (for greater discussion, see Section 6.2.2 of Xie et al., 2023, p. 413). Second, defining “transcription accuracy” can be a challenge (Baese-Berk et al., 2023). For the sake of simplicity, many studies (including the present experiment) employ a conservative, binary criterion for accuracy, even though certain types of incorrect responses are intuitively closer to the actual word than others (e.g., if the speaker said “pen,” incorrect responses of “pens” and “fruit” are not differentiated in most studies, even though “pens” is much more phonologically similar to the correct word than “fruit”). Third, sentences contain numerous phonological contrasts (Bradlow et al., 2023; Sidaras et al., 2009), which makes it difficult to manipulate heterogeneity while controlling for numerosity. This ultimately delimits the scope of the present work—although Section 1.2 presents numerosity and heterogeneity as possible theoretical accounts, the results do not rule out heterogeneity altogether.

If additional studies are conducted on apparent talker variability, they should ideally focus on different types of tasks. The general method of creating apparent talker variability (i.e., shifting F0 and formant frequencies through the Change Gender function in Praat) is not specifically contingent on speech-transcription-in-noise, so it could be useful to leverage other adaptation paradigms that have more narrowly defined response variables and provide greater insight into what contrasts are being learned (e.g., lexically guided perceptual learning, as in Reinisch & Holt, 2014; also, the infant phonetic discrimination literature is instructive here; Galle et al., 2015; Rost & McMurray, 2010).

#### 4.4.2 Exploring effects of speaker gender on L2-accent adaptation

Another caveat is that, by altering F0 and formant frequencies, the current experiment not only increases the number of perceived talkers in exposure, but also adjusts the apparent gender of these additional talkers. The mechanism of apparent talker variability thus comes with an important underlying assumption—that the gender of the exposure speakers is irrelevant in perceptual adaptation (e.g., if the novel test talker is male, generalization should still be greater after exposure to a group of mixed-gender speakers than after exposure to a single male talker). Research on the perception of L1-accent idiosyncrasies, however, suggests that speaker gender can play a critical role in adaptation, depending on which types of segments are heard. For instance, paralleling gender differences in speech production (Jongman et al., 2000; Kleinschmidt, 2019), Kraljic and Samuel (2007) observe stronger effects of gender for fricatives than stop consonants in perceptual adaptation (a thorough explanation of gender effects on adaptation is beyond the scope of the current paper; for a detailed review, see Kleinschmidt & Jaeger, 2015, pp. 179–180). These previous results for L1-accent adaptation lead to a specific prediction that can be tested in future work—apparent talker variability is more likely to successfully facilitate L2-accent generalization when the phonological contrast involves stop consonants (when gender is less relevant for adaptation), as opposed to fricatives (when gender is more relevant).

#### 4.4.3 On accent-independent adaptation and the integration of sociolinguistic methods

Only accent-specific adaptation has been discussed so far (i.e., conditions where the exposure and test talkers share the same language background). However, the theories presented in the current work are also applicable to other phenomena, such as accent-*independent* adaptation (Baese-Berk et al., 2013).

Using the same experimental paradigm as this study, Baese-Berk et al. (2013) compared comprehension of a novel Slovakian-accented English talker between three groups: (a) a *control condition* (exposure to five L1-English talkers); (b) a *single L2-accent condition* (exposure to five Mandarin-accented English talkers); (c) a *multiple L2-accent condition* (exposure to a Hindi-, Korean-, Mandarin-, Romanian-, and Thai-accented English talker). The third group ultimately showed the highest transcription accuracy, a finding that is well-explained by the ideal adapter framework (Kleinschmidt & Jaeger, 2015). Unlike participants who only heard a single L2-accent, listeners exposed to multiple L2-accents presumably picked up on the acoustic properties shared by all L2-accented speakers (as opposed to the properties just specific to Mandarin-accented English). This promoted the development of a broad, accent-independent model, thus facilitating perception of a talker from a novel language background (Slovakian).

An issue is that, similar to other work on talker variability (Raviv et al., 2022), studies of accent-independent adaptation are also typically confounded by numerosity and heterogeneity. Are listeners specifically attuning to the number of L2-accents in exposure, as predicted by a numerosity account? Or is the benefit of multiple-accent exposure a byproduct of its greater phonological variation compared with single-accent exposure, as proposed by a heterogeneity account? The latter claims that there is nothing special about the number of L2-accents itself—in theory, the effects of a multiple-accent exposure condition might be identical to a high acoustic variability, single-accent exposure condition.

To tease apart these possibilities, it could be helpful to manipulate the apparent identity of the talkers, building upon methods from sociolinguistic perception (Campbell-Kibler, 2010). One might envision a replication of Baese-Berk et al. (2013) where all listeners are exposed to identical stimuli, but given different information about the language background of the speakers prior to the experiment. For example, the participants might be placed in a multiple L2-accent exposure condition, with some told the genuine L1s of the speakers (e.g., Hindi, Korean, Mandarin, Romanian, and Thai) and others led to believe that all speakers share the same L1 (e.g., just Mandarin). If tested on a novel talker from a novel language background (e.g., Slovakian), a numerosity account would predict stronger generalization for the former group compared with the latter group, due to the *apparently greater* number of L2-accents in exposure. A heterogeneity account, by contrast, would predict no effect of this manipulation, since the actual acoustic properties of the exposure stimuli are kept the same across conditions.

In general, a growing literature has highlighted the importance of talker-indexical and socio-indexical cues on perceptual adaptation (e.g., Aoki & Zellou, 2023c; Tzeng et al., 2024; Zellou et al., 2023). More explicitly integrating sociolinguistic theory and methods into cognitive science remains a key step for future work.

## 5 Conclusion

Although the relationship between talker variability and L2-accent adaptation has been extensively studied, little to no work on L2-accent adaptation has examined the types of variation in the present study: apparent talker variability (the simulation of multiple talkers) and within-talker variation in speaking style. The current work contributes to our theoretical understanding of perceptual adaptation, showing that merely the *illusion* of multiple talkers can enhance cross-talker generalization, given sufficient acoustic similarity in speaking style.

## Notes

1. Recent work suggests that, contrary to findings on L2-accented speech, multiple-talker exposure may be necessary for generalization when adapting to highly unfamiliar, idiosyncratic phonetic shifts in L1-accented speech (e.g., a /p/ to [b] phonetic shift, where an L1-accented speaker produces words like “paint” as *baint*). For more details, see Aoki and Zellou (2025).

2. These speakers were part of a corpus of 96 talkers (half L1-English, half Mandarin-accented English), all of whom provided informed consent and completed the same recording procedure. The four specific talkers in this study were chosen because they all showed evidence of hyper-articulation in their hard-of-hearing-directed productions (see the acoustic analysis of Section 2.2 for details). The only reason for selecting male speakers is to be consistent with previous studies on L2-accent adaptation (e.g., Aoki & Zellou, 2023c; Baese-Berk et al., 2013; Bieber & Gordon-Salant, 2024; Bradlow & Bent, 2008; Xie et al., 2021; Xie & Myers, 2017).

3. The types of stimuli used in speech-transcription-in-noise experiments can vary from one study to the next. For instance, unlike the use of low-predictability sentences in the present work, certain studies employ semantically anomalous (Vaughn, 2019) or high-predictability sentences (Bradlow & Bent, 2008). In some cases, participants are asked to transcribe as many words as they can within each sentence (Van Engen et al., 2012), whereas other experiments instruct listeners to focus on sentence-final keywords (Clopper, 2011). The particular set of stimuli in this study were selected to align with the previous work of the authors (e.g., Aoki et al., 2022; Cohn et al., 2021). There is no immediately apparent theoretical reason for why the results reported here would not replicate with other kinds of sentences, although this must be examined through future research.

4. In casual speech, one of the speakers accidentally produced an extra affix on the final keyword of one of the sentences (“We’ve spoken about the *trucks*” instead of “We’ve spoken about the *truck*”). Only responses of “trucks” were considered correct for this particular stimulus, even though the original keyword was “truck.”

5. Thank you to Dr. Meghan Clayards for pointing out this possibility.

6. Since Bradlow and Bent (2008) originated the speech-transcription-in-noise paradigm, the list of studies in Table 3 was developed by sifting through all articles that cited Bradlow and Bent (2008) at the time of writing (*n* = 1,087 on July 1, 2025) and checking whether the methodology matched the two criteria listed in Section 4.3. Note that, to narrow the scope of the literature review, Table 3 underestimates the number of studies on L2-accent adaptation. Work on talker-specificity (exposure to a single talker) and work using nontranscription/repetition paradigms (e.g., lexically guided perceptual learning) have been excluded (Kim, Goldrick, M., & Bradlow, 2025; Reinisch & Holt, 2014). Space constraints also precluded Table 3 from containing more information, such as the gender of the talkers and the type of sentences used (e.g., low- vs. high-predictability).

7. The outsized presence of English in the literature (including the present study) should also be noted. As far as the authors are aware, all work using transcription or repetition tasks to study cross-talker generalization of L2-accented speech has been conducted in English, with only 3/18 studies recruiting listeners whose L1 was not American English (this issue at least partially seems to result from the usage of English-focused corpora). The pervasiveness of English is endemic to the cognitive sciences (Blasi et al., 2022), and more work is needed on cross-talker generalization in non-English contexts.
